# Failure to fire after an electrical injury – a complex syndrome in a soldier

**DOI:** 10.1186/s40779-015-0036-3

**Published:** 2015-03-28

**Authors:** FMH Ahmad, KVS Hari Kumar

**Affiliations:** Departments of Neurology & Endocrinology, Command Hospital, Chandimandir, India

**Keywords:** Complex regional pain syndrome, Bisphosphonates, Electrical injury, Regional osteoporosis

## Abstract

Complex regional pain syndrome (CRPS) is a disorder characterized by an intractable, disabling pain of the affected limb. It is triggered by various injuries and is often resistant to standard therapy. We report a young soldier with CRPS of the right hand sustained from an electrical injury, who had improvement in resting pain with Zoledronic acid. In this report, we discuss the therapeutic options and the role of bisphosphonates in CRPS.

## Background

Complex regional pain syndrome (previously termed reflex sympathetic dystrophy syndrome) is a condition that is characterized by pain, swelling, vasomotor changes, allodynia and regional osteoporosis. The disease is caused by a multitude of etiologies, including direct injury to nerves or limbs [1]. Complex regional pain syndrome (CRPS) is divided into two types based on the presence (CRPS II) or absence (CRPS I) of demonstrable nerve lesion. The pathophysiological basis of the disease is poorly documented, and it basically involves an aberrant host response to tissue injury [2]. The clinical heterogeneity of the disease is reflected in the resistance of this condition to the various available therapeutic options for the disorder.

Electrical injuries are common in clinical practice because of the accidental exposure to low voltage (<500 V) domestic circuits. The pattern of injuries differs based on the voltage (low or high), electricity source (lightning or electrical) and electricity type (alternating or direct) [3]. The acute effects of electrical injuries are burns, cardiac arrhythmias, myoglobinuria and sudden cardiorespiratory arrest [4]. Delayed neurological manifestations, including CRPS, predominate during the chronic phase after an electrical injury [5]. We recently encountered a soldier who reported to us with features of CRPS after sustaining a domestic electrical injury that improved with bisphosphonates. We report this case to highlight an unusual complication and the role of bisphosphonates in the treatment of resistant cases of CRPS.

## Case presentation

**Figure 1:**
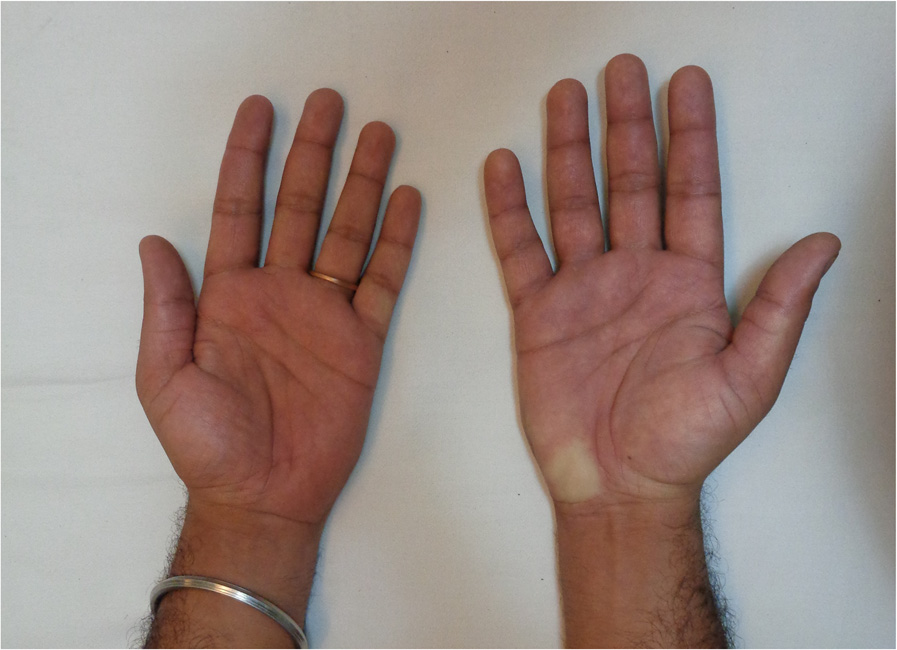
**Swollen right hand showing hyperemia.**

A 34-year-old man was referred to our hospital in May 2014 with a history of pain and swelling of the right hand for a duration of 3 months. The patient sustained an electrical shock to the right hand while repairing a domestic electrical appliance. He denied any cutaneous injury, loss of consciousness or injury to any other body part due to the electrical shock. He noticed pain in his right hand within one week of injury, which was treated with analgesics without relief. He noticed a subsequent swelling, deep aching pain, cutaneous hypersensitivity and discoloration of the right hand. The patient denied aggravation of symptoms during exposure to cold or any diurnal variation of the symptoms. The patient indicated severe pain, which he rated as 9 on a 10-point visual analogue scale (VAS). He was treated with a short course of oral prednisolone with physiotherapy at a peripheral hospital, but the improvement was not significant. The patient is employed as an infantry soldier, and the condition prevented him from being able to handle any weapons. He was also unable to use his right hand for activities of daily living. The patient denied alcohol consumption or tobacco use, and his previous medical history was unremarkable. Examination revealed stable vital parameters, and systemic examination was unremarkable. His right hand was markedly tender, hyperemic with diffuse swelling and cutaneous hypersensitivity (Figure 1). Other vasomotor changes were absent, and the radial pulse was normally palpable on the right side. Muscle atrophy was not apparent, and there was generalized weakness of the hand muscles, likely because of the reduced effort by the patient.

**Figure 2:**
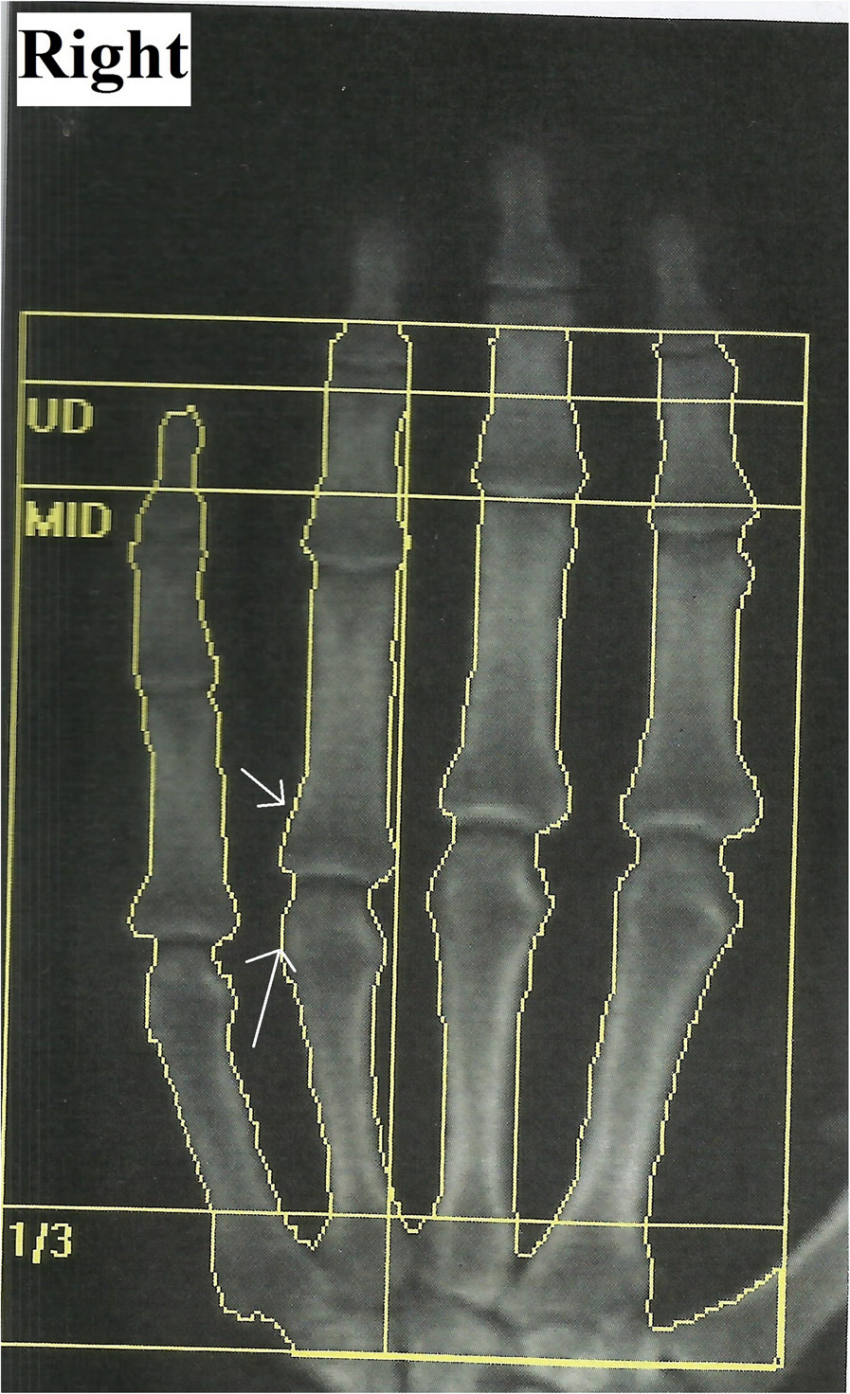
**DEXA scan showing regional osteoporosis of right hand bones with juxta articular osteopenia (arrows).**

His hematological and biochemical parameters were all normal, including the C-reactive protein, creatine kinase, calcium, phosphorus, alkaline phosphatase and 25-hydroxy vitamin D levels. A nerve conduction study and electromyography of the left upper extremity showed no evidence of neuropathy or myopathy. A Dual Energy X-ray Absorptiometry (DEXA) scan revealed a significant reduction in bone mass of the right side (0.47 g/cm^2^) compared to the left (1.1 g/cm^2^) side (Figure 2). He was diagnosed as a case of CRPS type I based on the clinical features, and he was treated with Zoledronic acid (4 mg, intravenously once monthly). Zoledronic acid was considered the best option in our patient because of the failure of conventional therapies, including analgesics, glucocorticoids and physiotherapy. He experienced partial pain relief over the next 7 days, and he rated the current pain as 5/10 on the VAS. He was given a repeat injection of Zoledronic acid after one month, and the pain during the last follow-up was reduced to 2/10 VAS with a complete reduction of the swelling. He started handling and firing fire arms last month, but he is unable to use his right arm completely for all activities. Currently, the patient has no resting pain, and he is able to use the right hand for all activities of daily living. However, the patient has mild pain during prolonged use of the hand, and he is unable to lift heavy objects using the right hand. We did not evaluate the upper hand disability initially with an objective assessment for comparison after treatment. Currently, the patient has no resting pain, but he has minimal pain with prolonged use of the limb muscles. A repeat DEXA scan at the end of 3 months revealed minimal improvement in bone mass (0.78 g/cm^2^) on the right side.

## Discussion

Complex Regional Pain Syndrome (CRPS) is a disorder that is characterized by intense pain in association with sensory, motor, autonomic and trophic abnormalities [1]. A variety of pathophysiological insults trigger CRPS, and the condition is often resistant to therapy, which necessitates a multimodal approach. Electrical injuries are rarely reported as an etiological factor in CRPS [3]. Electrical injuries result in neurological damage during acute and chronic stages. Acute effects include paralysis, neuropathy and chronic effects, such as vertigo, tinnitus, depression and tremors [6]. The DASH (Disabilities of the Arm, Shoulder and Hand) questionnaire is a self-reported measure to assess disabilities that are specific to the upper extremities [7]. Unfortunately, we did not assess disability using the DASH scale prior to the Zoledronic acid therapy, which limited an objective assessment. Previous reports suggest that more than half of CRPS patients have a persistent long-lasting disability despite therapy [8]. Our patient exhibited a persistent impairment in function, but his resting pain was relieved after therapy.

Electrical injury rarely results in CRPS, and this type of injury mostly leads to CRPS II rather than CRPS I [9]. CRPS I and II are neuropathic conditions, but the lack of demonstrable neuropathy on nerve conduction study in our patient precluded the diagnosis of CRPS II. Our patient did not exhibit features of myopathy on the EMG. Therefore, we did not consider the possibility of a direct muscle injury from the electrical insult. The use of EMG is fraught with limitations of availability and interpretation. Assessment for muscle atrophy is performed using clinical (measurement of girth) and radiological (ultrasonography) methods. Subclinical muscular atrophy is identified by a reduced bulk and increased echogenicity of muscles on ultrasonography [10]. Nerve and muscle injury may have contributed to the muscle atrophy after an electrical shock. Unfortunately, we did not perform ultrasonography of the muscle to identify subclinical muscular atrophy. The pathophysiological basis of CRPS is poorly understood, and the disease is characterized by a high turnover of bone and regional bone loss. The disease is caused by neurogenic inflammation, nociceptive sensitization, vasomotor dysfunction and maladaptation of neuroplasticity [1]. Other novel pathogenic mechanisms are implicated in CRPS, including the formation of multiple myofascial trigger points and the intermittent release of inflammatory mediators due to repeated movement [11,12].

CRPS is not easily amenable to therapy despite the availability of multiple therapeutic modes. The therapeutic approach involves the use of nonsteroidal anti-inflammatory drugs, narcotic analgesics, corticosteroids, calcitonin, serotonin reuptake inhibitors, bisphosphonates and sympathetic blocks [2]. The patients often require multiple therapeutic modalities of therapy, and CRPS may persist in a majority of patients despite the use of all remedial measures. Fortunately, our patient showed significant resolution of his symptoms after Zoledronic acid, which limited the necessity of other therapeutic options. Few investigators have used neuromodulation techniques, such as stimulation of peripheral nerves, spinal cord, motor cortex, and deep brain, and intrathecal drug delivery systems in resistant cases of CRPS [13]. Bisphosphonates potently inhibit bone turnover, and these drugs are useful in all cases of CRPS I [14]. A previous trial using intravenous neridronate in a similar situation of early CRPS showed a >50% reduction in the pain VAS score in 73% of patients versus 32% in the placebo group [15]. The reduction of pain is associated with decreased allodynia, hyperalgesia and improvements in pain-related quality of life indices. Bisphosphonates relieve the pain associated with CRPS by reducing the osteoclast activity and the release of nociceptive mediators. The term “Orphan medicinal products” is used for drugs that are under evaluation and may be useful in rare disorders based on their physiological actions. Zoledronic acid has been given an orphan designation in the United States, but not in the European Union, for its use in CRPS [16].

## Conclusion

We report a patient with CRPS I who presented after an electrical injury and showed a good response to the use of intravenous Zoledronic acid. Our report highlights the beneficial role of bisphosphonates in the few CRPS patients who are resistant to standard therapy.

## Consent

Written informed consent was obtained from the patient for publication of this Case Report and any accompanying images. A copy of the written consent is available for review by the Editor-in-Chief of this journal.

## References

[1] Marinus J, Moseley GL, Birklein F, Baron R, Maihöfner C, Kingery WS (2011). Clinical features and pathophysiology of complex regional pain syndrome. Lancet Neurol..

[2] Borchers AT, Gershwin ME (2014). Complex regional pain syndrome: a comprehensive and critical review. Autoimmun Rev..

[3] Jost WH, Schönrock LM, Cherington M (2005). Autonomic nervous system dysfunction in lightning and electrical injuries. NeuroRehabilitation..

[4] Duff K, McCaffrey RJ (2001). Electrical injury and lightning injury: a review of their mechanisms and neuropsychological, psychiatric, and neurological sequelae. Neuropsychol Rev..

[5] Kim CT, Bryant P (2001). Complex regional pain syndrome (type I) after electrical injury: a case report of treatment with continuous epidural block. Arch Phys Med Rehabil..

[6] Freund W, Wunderlich AP, Stuber G, Mayer F, Steffen P, Mentzel M (2010). Different activation of opercular and posterior cingulate cortex (PCC) in patients with complex regional pain syndrome (CRPS I) compared with healthy controls during perception of electrically induced pain: a functional MRI study. Clin J Pain..

[7] Hudak PL, Amadio PC, Bombardier C (1996). Development of an upper extremity outcome measure: the DASH (disabilities of the arm, shoulder and hand) [corrected]. The Upper Extremity Collaborative Group (UECG). Am J Ind Med.

[8] deMos M, Huygen FJPM, van der Hoeven-Borgman M, Dieleman JP, Stricker BHC, Sturkenboom MCJM. Outcome of the complex regional pain syndrome. Clin J Pain. 2009;25:590–97. 10.1097/AJP.0b013e3181a11623 19692800

[9] Cohen JA (1995). Autonomic nervous system disorders and reflex sympathetic dystrophy in lightning and electrical injuries. Semin Neurol..

[10] Vas L, Pai R, Menon M. Ultrasound appearance of forearm muscles in eighteen patients with complex regional pain syndrome type −1 of the upper extremity. Pain Practice. 2013;13:76–88. 10.1111/j.1533-2500.2012.00539.x 22494480

[11] Vas L, Pai R (2012). Successful reversal of complex regional pain syndrome type 1 of both upper extremities in five patients. Letter, Pain Medicine..

[12] Vas L, Pai R (2014). Reversal of complex regional pain syndrome - type 2, and the subsequent management of complex regional pain syndrome – type 1 occurring after corrective surgery for residual ulnar claw. Pain Med.

[13] Levy RM (2012). Evidence-based review of neuromodulation for complex regional pain syndrome: a conflict between faith and science?. Neuromodulation..

[14] Schott GD (1997). Bisphosphonates for pain relief in reflex sympathetic dystrophy?. Lancet..

[15] Varenna M, Adami S, Rossini M, Gatti D, Idolazzi L, Zucchi F (2013). Treatment of complex regional pain syndrome type I with neridronate: a randomized, double-blind, placebo-controlled study. Rheumatology (Oxford)..

[16] Littlejohn G (2013). Therapy: Bisphosphonates for early complex regional pain syndrome. Nat Rev Rheumatol..

